# Poly(curcumin β-amino ester)-Based Tablet Formulation for a Sustained Release of Curcumin

**DOI:** 10.3390/gels8060337

**Published:** 2022-05-30

**Authors:** Vinod S. Patil, Benjamin C. Burdette, J. Zach Hilt, Douglass S. Kalika, Thomas D. Dziubla

**Affiliations:** Department of Chemical and Materials Engineering, University of Kentucky, Lexington, KY 40506, USA; patil.vinod2007@gmail.com (V.S.P.); benburdette9@gmail.com (B.C.B.); zach.hilt@uky.edu (J.Z.H.); douglass.kalika@uky.edu (D.S.K.)

**Keywords:** Poy(β-amino ester), curcumin, hydrogels, tablets, solubility, amorphous, oral dosage form, crosslinked systems, controlled drug delivery, prodrug

## Abstract

Oral drug delivery remains the most common and well tolerated method for drug administration. However, its applicability is often limited due to low drug solubility and stability. One approach to overcome the solubility and stability limitations is the use of amorphous polymeric prodrug formulations, such as poly(β-amino ester) (PBAE). PBAE hydrogels, which are biodegradable and pH responsive, have shown promising results for the controlled release of drugs by improving the stability and increasing the solubility of these drugs. In this work, we have evaluated the potential use of PBAE prodrugs in an oral tablet formulation, studying their sustained drug release potential and storage stability. Curcumin, a low solubility, low stability antioxidant drug was used as a model compound. Poly(curcumin β-amino ester) (PCBAE), a crosslinked amorphous network, was synthesized by a previously published method using a commercial diacrylate and a primary diamine, in combination with acrylate-functionalized curcumin. PCBAE-based tablets were made and exhibited a sustained release for 16 h, following the hydrolytic degradation of PCBAE particles into native curcumin. In addition to the release studies, preliminary storage stability was assessed using standard and accelerated stability conditions. As PCBAE degradation is hydrolysis driven, tablet stability was found to be sensitive to moisture.

## 1. Introduction and Background

Oral drug formulations remain the most common and patient preferred form of drug delivery. Oral administration is used for treating a wide range of diseases localized to the gastrointestinal (GI) tract, such as gastritis, enterocolitis, enteritis, inflammatory bowel diseases, as well as for delivering drugs to the whole body for systemic disorders [1,2,3,4]. However, the efficacy of oral formulations is often limited by poor stability in the acidic stomach environment and by low solubility of the drug. The drug stability can usually be improved by using enteric coatings, which rely on pH-dependent swelling for drug release [5]. However, enteric coatings can often be ineffective if the patient is taking proton pump inhibitors, which result in increased stomach pH, or when the coatings are broken [6]. In addition, the solubility of drug is typically improved through the creation of a salt form, or the development of an amorphous dispersion or a prodrug formulation [7,8,9]. In order to create a salt form, the drug must have appropriate acidic or basic groups, and it can often be challenging to find appropriate counter ion pairs [10]. On the other hand, amorphous dispersions can face processing challenges, due to their inherent limited thermal stability [11]. Amorphous dispersions are also limited in drug loading since at high drug loadings, phase separation and drug crystallization can occur [12,13].

Antioxidant delivery to the lower GI tract is vital since inflammatory bowel diseases, such as ulcerative colitis, infectious colitis, or ischemic colitis, are caused by the formation and cascade of reactive oxygen species that lead to a chronic inflammatory response [3,14,15]. The resulting oxidative stress can be overcome through use of antioxidants, which can scavenge the reactive oxygen species, reducing oxidative stress signaling, and thus allowing the tissue to heal and reverse inflammation [16,17,18]. Different approaches have been explored for colon-specific delivery that include enzymatic degradation of polymers such as guar gum, polymers with pH-dependent solubility, timed release systems and osmotically controlled release systems [5,19,20,21]. While all promising, each possesses limitations that hamper their applicability. For instance, in the case of guar gum-based tablets, a seven-day enzyme induction period is needed before actually using the tablets. In addition, as the pH of the gastrointestinal tract is known to vary under different conditions and with the use of proton pump inhibitors, it may be difficult to deliver drugs to the desired site based solely upon the pH-dependent solubility of the polymer excipients [22]. Similarly, due to the considerable variation in transit time through the GI tract, timed release systems do not always work, while the manufacturing process of osmotically controlled release systems can be complex and expensive [23]. Due to these considerations, a system with a combination of pH-dependent and sustained release would be desired for providing a better strategy to orally deliver poorly soluble drug molecules. Such a system would need to have very slow to no degradation at low pH and a relatively controlled rate of degradation at higher pH to ensure that there is no significant degradation during the typical stomach transit time of 30 min to 4 h.

One possible way to overcome these limitations is the use of a new amorphous polymeric prodrug hydrogel, i.e., poly(β-amino ester) (PBAE), in a tablet formulation. Instead of physically dispersing the drug molecule in the polymer domain, which is used in amorphous dispersions, a drug molecule of interest is chemically incorporated into the hydrogel network through covalent bonding. This improves the stability of the drug by hindering motion of the labile groups on the molecule and by limiting the access of degradative agents to these groups. Once in contact with water inside the body, PBAE undergoes hydrolytic degradation to release the drug in its original form [24,25]. While the degradation of PBAE is pH dependent, the rate of degradation of amine containing PBAEs is relatively slow at lower pH [26,27]. Due to this unique pH response, only minimal degradation occurs in the upper gastrointestinal tract, with degradation starting upon reaching the higher pH environments of the lower GI tract. Even though PBAE starts degrading at higher pH, this degradation is not instantaneous and occurs over a time period based on the PBAE formulation. Since the drug is covalently bonded to the polymer network, dose dumping, typically observed for other formulation strategies, is completely avoided. Additionally, induction treatments and enteric coatings are eliminated with the use of PBAEs, making the crosslinked polymer network an advantageous drug delivery system that can provide the improved drug stability and controlled release of drugs upon the hydrolytic degradation of polymers that can be tailored for different release times and drug loadings.

In this study, we have developed an oral PBAE hydrogel-based formulation for the delivery of curcumin, which is a hydrophobic drug. Curcumin is a polyphenolic compound found in the Indian food spice turmeric, which has antioxidant and anti-inflammatory properties. Poly(curcumin β-amino ester) (PCBAE) hydrogels, with release times ranging from 5 to 25 h, have been developed previously by tuning the hydrophobic composition of the network [28,29], and in vitro studies have shown PCBAE to be non-toxic [24]. Herein, we have investigated an oral PCBAE tablet formulation, which contains the PCBAE hydrogel, specifically selected based on degradation time, and commonly used oral solid dosage excipients, *viz*., microcrystalline cellulose (MCC) and magnesium stearate. The tablets were evaluated for sustained curcumin release, as well as overall storage stability. Sustained curcumin release is obtained due to the hydrolytic degradation of PCBAE, which releases curcumin back in its original form. The results of detailed release studies are presented for the as-prepared PCBAE hydrogel films, PCBAE hydrogel microparticles, and the tablet formulation exposed to a range of controlled storage conditions.

## 2. Results

### 2.1. PCBAE Hydrogel Film Degradation and Tablet Dissolution

**Figure 1:**
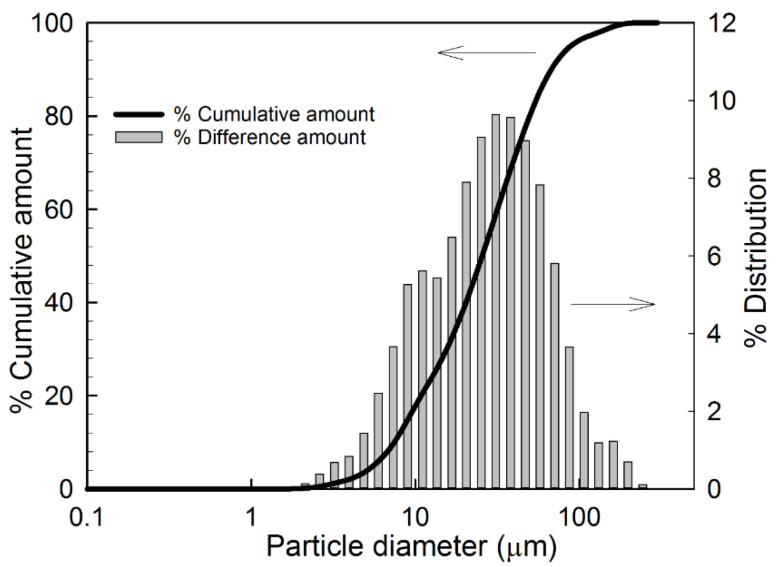
Particle size distribution for 90:10 CMA:PEG400DA PCBAE microparticles analyzed using Shimadzu SALD-7101 nanoparticle size analyzer. THE D_50_ for these particles was 28 µm.

The 90:10 CMA:PEG400DA hydrogel film was selected for making the microparticles used to prepare the prodrug tablet. The corresponding theoretical drug loading in the film was 52.7% (weight of curcumin/weight of hydrogel film). Particle size analysis of the cryo-milled 90:10 CMA:PEG400DA gel powder was performed using the Shimadzu nanoparticle size analyzer by suspending the microparticles in PBS. The particle size distribution data for these particles are presented in Figure 1. The D_50_ for these particles was 28 µm.

**Figure 2:**
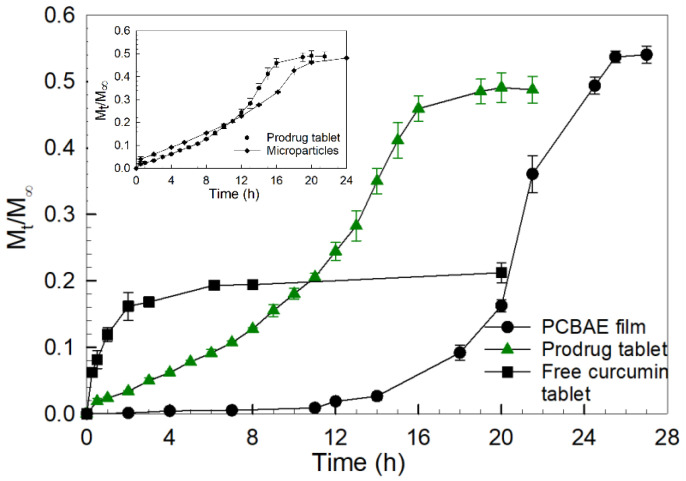
Curcumin release profiles for 90:10 CMA:PEG400DA PCBAE hydrogel film and tablets containing PCBAE microparticles (i.e., prodrug tablet) and free curcumin powder, respectively, in phosphate buffered saline (PBS) with 0.1 % SDS at 37 °C, using USP apparatus II and analysis using the UV–Vis method. Inset plot shows curcumin release for prodrug tablet vs 90:10 CMA:PEG400DA as prepared microparticles without excipients.

In Figure 2, the curcumin release profile from the hydrolytic degradation of the hydrogel film with a ratio of 90:10 CMA:PEG400DA, along with the curcumin release profile for the (microparticle-containing) prodrug tablet and free curcumin tablet in PBS (pH 7.4) are presented. For the first 12 h of film degradation, very little drug release is observed for the PCBAE film. However, after 12 h, the polymer starts to display significant swelling as the network crosslinks start breaking down due to the hydrolysis of ester bonds [28], which leads to curcumin release from the hydrogel film. A sustained curcumin release up to approximately 26 h is observed from the hydrogel film.

The PCBAE prodrug tablet underwent quick disintegration, thereby releasing MCC and PCBAE gel particles, followed by hydrolytic degradation of the PCBAE gel particles. Since curcumin is covalently bonded in the polymer network and unreacted monomers were removed during the washing step, no initial burst release of curcumin is observed during the degradation of these gels. This is one of the key advantages of the PCBAE-based prodrug approach, since initial burst release is a common problem encountered in the traditional approach of physically entrapping drug molecules in a polymer network [30]. Here, curcumin is released from PCBAE microparticles only after the hydrolytic cleavage of the ester bonds. Curcumin release from the microparticles was assessed by measuring the absorbance at 420 nm. A sustained curcumin release is observed over about 16 h. The inset plot in Figure 2 shows the curcumin release from the as-prepared microparticles compared to the release measured for the prodrug tablet. This plot shows that the excipients used in tablet formulation do not affect the curcumin release profile and that the release was governed solely by microparticle (i.e., hydrogel) degradation.

In a similar way to the prodrug tablet, tablets compounded with “free” curcumin powder and dose matched with the prodrug tablet demonstrated quick disintegration in PBS. An almost immediate curcumin release was observed with the free curcumin tablet, as compared to the controlled release of curcumin that was observed for the PCBAE prodrug tablet (refer Figure 2). The PCBAE prodrug tablet displayed a final M_t_/M_∞_ value of 0.5, which is 2.5 times the value obtained for the free curcumin tablet (M_t_/M_∞_ value of 0.2), demonstrating improved apparent solubility over free curcumin with the same dissolution conditions. A higher M_t_/M_∞_ value is desired, as it represents higher drug recovery as well as higher in vitro drug solubility in PBS. The M_t_/M_∞_ value for the free curcumin tablet is significantly lower than that of the PCBAE, due to the crystalline character of hydrophobic curcumin, which limits its dissolution and results in the presence of solid curcumin precipitate in the PBS medium. Poor solubility is a concern with many hydrophobic crystalline drugs, such as indomethacin, nifedipine, and griseofulvin, and amorphous dispersions of these drugs are often explored to enhance their dissolution [31,32,33,34]. The PCBAE networks are amorphous in nature due to the presence of crosslinks that prevent crystallization of the curcumin. As the polymer degrades, curcumin is released in its original form and does not undergo crystallization as the curcumin domain is too small to form any crystal nuclei [12]. Additionally, PEG-based degradation products of PCBAE appear to enhance the solubility of the released curcumin.

**Figure 3:**
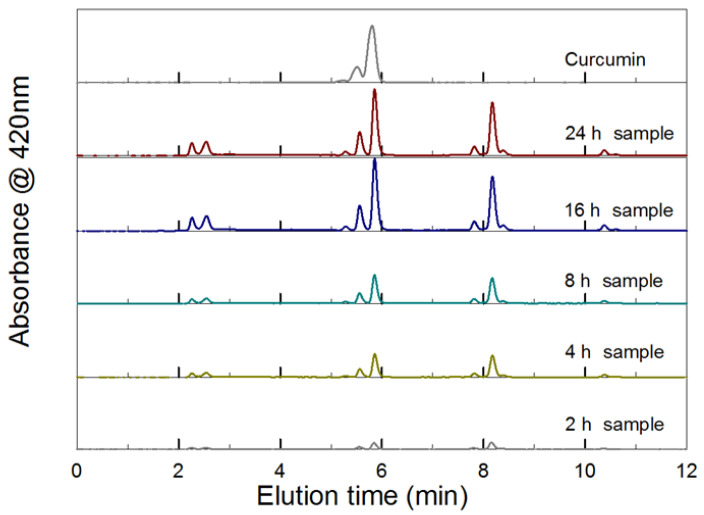
HPLC peak elution profile (420 nm) for supernatant samples collected during aqueous dissolution of the PCBAE prodrug tablet.

The release of native curcumin was confirmed by HPLC analysis of selected aliquots collected during the dissolution study of the PCBAE prodrug tablet, as shown in Figure 3. Early elution peaks between 2-3 min represent hydrophilic components, which could be PEG curcumin adducts. A continuous release of curcumin (HPLC elution time between 5.2 and 6 min) is observed with an increase in degradation time. Along with curcumin, curcumin monoacrylate (elution time between 7.6 and 8.5 min) and a small amount of curcumin diacrylate (elution time between 10 and 10.8 min) are also recovered from the HPLC column. The individual species of the curcumin acrylation mixture have been identified by liquid chromatography-mass spectrometry (LCMS), as reported in an independent study [35].

### 2.2. Tablet Storage Stability Study

**Figure 4:**
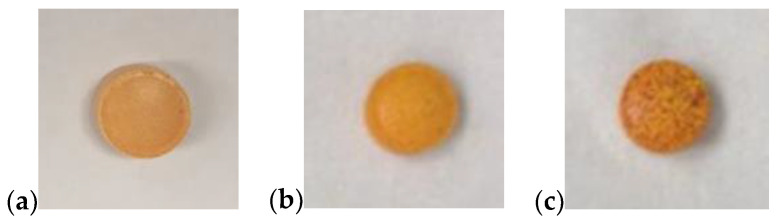
PCBAE prodrug tablets: (**a**) T0 (initial); (**b**) one week at standard storage conditions, i.e., 25 °C and 57% RH; (**c**) one week at accelerated storage conditions, i.e., 40 °C and 75% RH.

**Figure 5:**
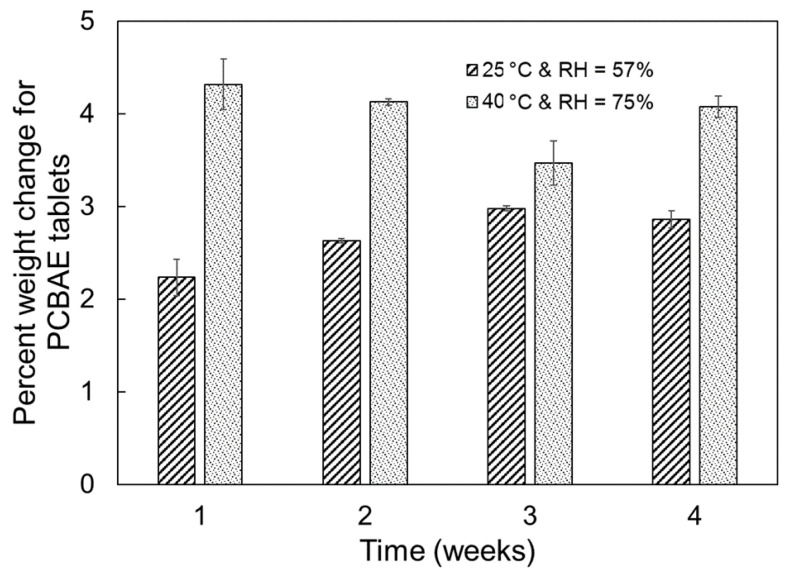
PCBAE prodrug tablet moisture absorption as a function of storage time for standard and accelerated storage conditions. Percent weight change from T0 (initial) tablet weight.

To study the stability of the tablet formulations according to USP protocol, the tablets were stored under standard and accelerated storage conditions. The tablets were stored in open conditions as a worst-case scenario to allow assessment of direct exposure to moisture. The effect of storage conditions on tablet stability was assessed by comparing the moisture absorption and curcumin release profiles for tablets with varying storage exposure history. Images of an as-prepared tablet and tablets after storage at standard and accelerated conditions for 1 week are presented in Figure 4. The tablets stored at accelerated conditions exhibited a more intense orange surface coloration compared, to the as-prepared tablet and tablets stored at standard conditions. The weights of the tablets were measured before and after storage at standard and accelerated conditions to quantify the moisture absorption of the tablets. The percentage weight change due to moisture absorption is shown in Figure 5. The tablets that were stored at the accelerated conditions had higher moisture absorption than those stored at standard conditions for a given exposure time. Furthermore, overall moisture absorption increased with longer storage times at the standard condition, while tablets stored at the accelerated condition reached equilibrium weight within a week.

**Figure 6:**
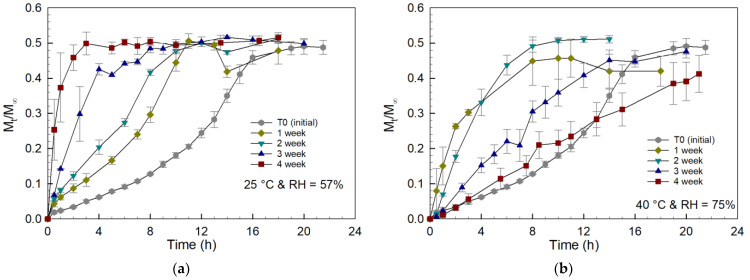
Curcumin release profiles from dissolution of PCBAE prodrug tablets stored at (**a**) standard conditions of 25 °C and RH = 57%; (**b**) accelerated conditions of 40°C and RH = 75% with analysis using UV–Vis method.

Aqueous dissolution studies were performed on all the tablets that were stored at standard and accelerated storage conditions for different times. The curcumin release data for these studies are reported in Figure 6. The time needed for total curcumin release from the prodrug tablets stored at standard storage conditions decreased as the storage time was increased, with tablets stored for 4 weeks showing a total release time of just 1 h. All the tablets stored at standard conditions disintegrated within the first two hours of the dissolution study, followed by the release of curcumin from PCBAE microparticles that had undergone partial degradation during the storage.

For the accelerated storage conditions, the tablets stored for 1 week had the fastest curcumin release rate. The overall curcumin release time increased as exposure time was increased, with the tablets stored for 4 weeks requiring 24 h for complete release. This delay in curcumin release for the tablets stored over longer times was not anticipated and appears to reflect a change in the physical disintegration dynamics of these tablets, as compared to those samples stored under standard conditions. Specifically, for the tablets that were stored at accelerated conditions, the observed disintegration times in PBS were 4 h, 8 h, 14 h and 20 h for storage times of 1 week, 2 weeks, 3 weeks and 4 weeks, respectively. These results were in direct contrast to the behavior observed for the tablets held at standard conditions, which all disintegrated within two hours regardless of prior storage history. The time needed for total curcumin release from the tablets stored at accelerated conditions correlated closely with the disintegration time of each tablet, suggesting that the delayed physical disintegration of the tablets was the controlling mechanism for curcumin release for this series of tablets. However, even though the as-prepared tablets and tablets held at standard and accelerated conditions had different degradation times, all of them reached the same plateau M_t_/M_∞_ value with a final curcumin concentration corresponding to 21.5 μg/mL (based on UV–Vis measurement), suggesting that only the dissolution profile was impacted.

**Figure 7:**
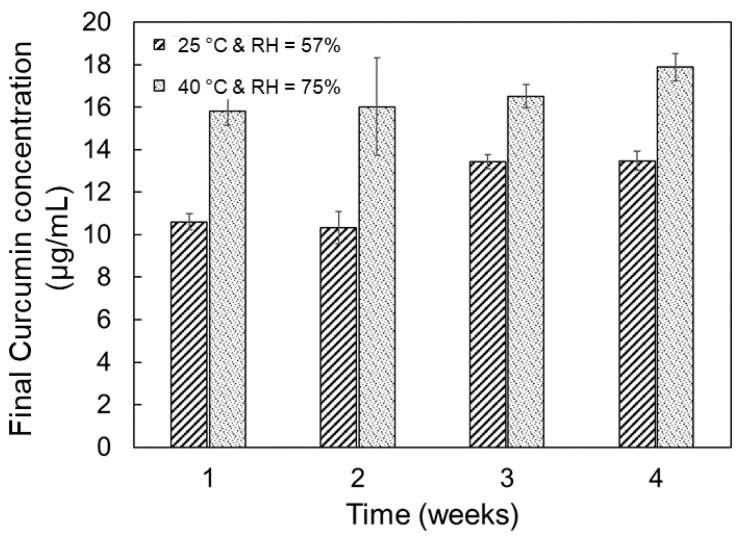
Free curcumin concentration based on HPLC analysis (elution between 5 and 6 min) of the final degradation supernatants collected from the dissolution of PCBAE prodrug tablets stored at standard and accelerated conditions.

The HPLC elution curves for selected release time points are presented in Figure 3. An analysis of the elution curves for the final release time points was performed to determine the concentration of curcumin in the degradation products obtained for each tablet storage history; these data are reported in Figure 7. The concentration of curcumin for the 100% theoretical release based on the drug loading of the polymer would be 42.2 μg/mL, indicating only a 25% to 40% recovery of pure curcumin from the dissolved tablets. These lower values reflect the presence of acrylated curcumin and PEG-curcumin adducts in the degradation products from the PCBAE network. Based on the HPLC analysis, a higher overall curcumin concentration is indicated for the tablets exposed to the accelerated storage conditions compared to standard conditions. For the standard conditions, a modest increase in curcumin concentration is observed with increasing prior storage time.

**Figure 8:**
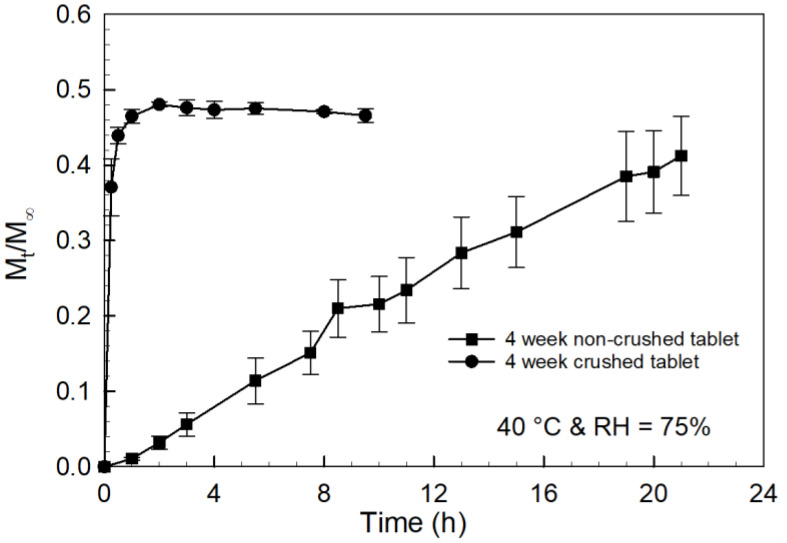
Curcumin release profiles for PCBAE prodrug tablets stored at accelerated conditions for 4 weeks with analysis using UV–Vis method. Crushed tablet compared with a non-crushed tablet.

To further understand the delay in the release observed for the tablets stored at the accelerated conditions (40 °C and RH = 75%), another set of PCBAE prodrug tablets was stored at the same conditions, and the tablets were then crushed into powder before adding to the dissolution apparatus. The curcumin release curves obtained from the dissolution of these two sets of tablets are shown in Figure 8. It was observed that essentially, the entire curcumin release from the crushed tablets occurred within the first hour, whereas at least 20 h was required for full curcumin release from the uncrushed tablets. This result confirmed that the PCBAE microparticles present in the prodrug tablets degraded extensively to curcumin during the 4 weeks of storage at accelerated conditions and that an increase in the time needed for total curcumin release in the uncrushed tablets primarily reflects the difference in the physical disintegration characteristics of the tablets, as noted above.

**Figure 9:**
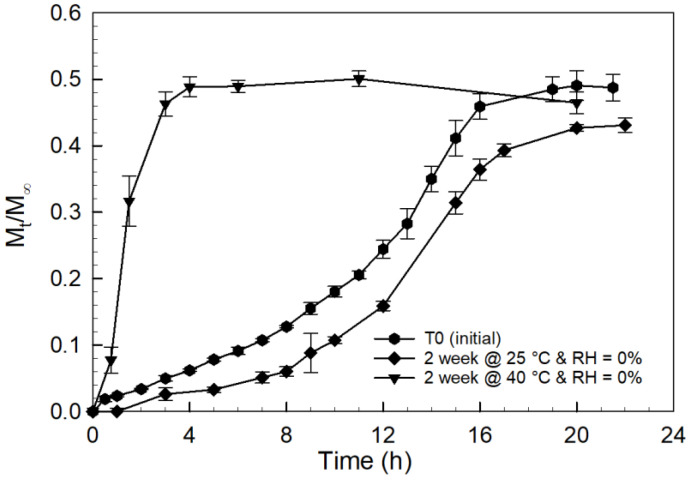
Curcumin release profiles for PCBAE prodrug tablets stored at 25 °C and 40 °C for 2 weeks in the absence of surrounding moisture with analysis using UV–Vis method.

The effect of temperature on tablet stability in the absence of surrounding moisture was analyzed by storing the tablets in a desiccant chamber (RH = 0%) for 2 weeks at 25 °C and 40 °C; the corresponding curcumin release curves for these PCBAE prodrug tablets are presented in Figure 9. The curcumin release profile for the tablets stored at 25 °C was not significantly affected considering experimental error for a non-validated synthesis process; however, the tablets stored at 40 °C showed total curcumin release within 3 h. This suggests that even in the absence of ambient moisture, a small amount of moisture present in the MCC (~3–5%) binder contributes to an increased rate of hydrolytic degradation of the microparticles at elevated temperatures.

**Figure 10:**
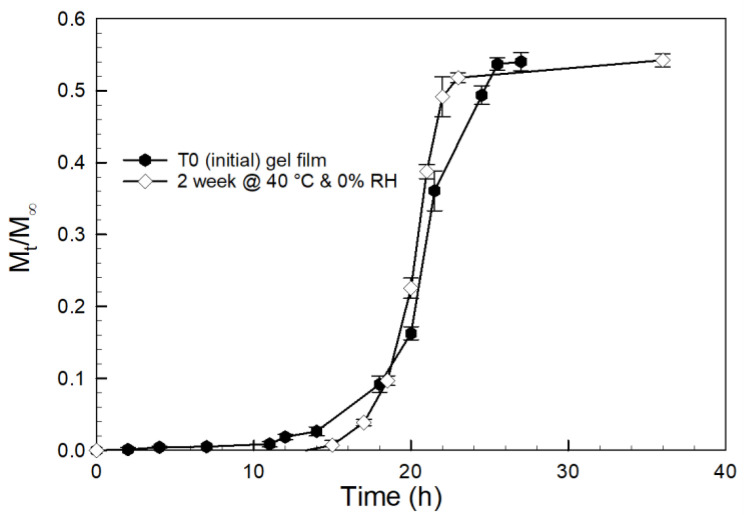
Curcumin release profiles in PBS for 90:10 CMA:PEG400DA PCBAE hydrogel films as-prepared at ambient conditions, and stored at 40 °C for 2 weeks in the absence of moisture (i.e., 0% RH) with analysis using UV–Vis method.

To further verify the influence of inherent moisture on the storage stability of the tablet formulation, as-prepared gel films were stored at 40 °C for 4 weeks in a desiccant chamber (RH = 0%). The curcumin release profile for the degradation of these gels is reported in Figure 10. For the gel films, no degradation was observed at 40 °C in the absence of surrounding moisture, which was the opposite of the release profile for the tablets stored at the same conditions (refer Figure 9). This confirms that the small amount of moisture present in the MCC binder in the prodrug tablet is sufficient to cause the degradation of PCBAE at 40 °C, with a dramatic impact on the resulting curcumin release properties.

### 2.3. Antioxidant Activity

**Figure 11:**
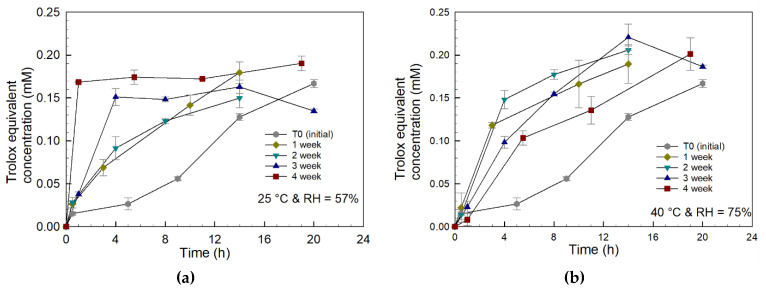
Antioxidant capacity of tablet dissolution supernatants measured for all storage times at standard conditions using Trolox equivalent antioxidant capacity (TEAC) in-vitro antioxidant measurement assay. (**a**) Standard conditions of 25 °C and RH = 57%; (**b**) accelerated conditions of 40 °C and RH = 75%.

Selected degradation samples were analyzed for their functional activity using the TEAC assay, which measures antioxidant activity using Trolox as a reference standard [36]. The TEAC activities in the release studies for prodrug tablets stored at both standard and accelerated storage conditions are presented in Figure 11 and show data trends similar to the curcumin release profiles, as reported in Figure 6. The TEAC results confirmed that the curcumin released during the tablet dissolution studies maintains antioxidant activity. The final equivalent Trolox concentration for all the curves is consistent with the final curcumin release concentration, as determined by UV spectroscopy in Figure 6.

## 3. Discussion

In this work, PCBAE microparticle-based prodrug tablets were synthesized for the controlled release of curcumin with improved dissolution properties. The advantage of this formulation is that the solubility of the drug is improved due to the crosslinked amorphous structure of the PCBAE and the stability of the drug is improved through the protection of labile groups via the covalent conjugation of curcumin within the polymer network. The controlled release of curcumin is achieved through the hydrolytic degradation of PCBAE when it encounters water. The curcumin is released following the degradation of the amorphous PCBAE and does not crystallize due to the lower local concentration of curcumin as well as the stabilization provided by the oligomeric degradation products of PCBAE, thereby improving curcumin solubility. A similar approach has been used to develop quercetin PBAE nanogels for the treatment of cellular oxidative stress [37]. The PCBAE system is advantageous for oral delivery as the drug is protected from the low pH environment present in gastric fluid, due to the lower rate of polymer degradation at lower pH, while it degrades at a controlled rate at higher pH [26]. PBAEs undergo faster degradation at higher pH since the amine can act as an intramolecular nucleophilic catalyst and expedite the hydrolysis of PBAE [26,27]. Apart from this, the carboxylic acid products formed from the hydrolysis of PBAE are neutralized in higher pH surroundings, driving further hydrolysis. The pH response was also confirmed for PCBAEs, where it degraded faster at pH 7.4 than at pH 1.5 (refer. Figure S1).

It was determined from a previous study that PCBAE hydrogels with 90:10 CMA:PEG400DA composition degraded over 25 h [28]. In addition to this, among the samples studied, the 90:10 composition had the highest glass transition temperature (T_g_ = 67 °C), which reflects a relatively favorable overall stability of the network. For these reasons, PCBAE films with the 90:10 CMA:PEG400DA composition were used for the tablet formulations characterized in this work.

### 3.1. Difference in Curcumin Release Profile for PCAE Film Versus PCBAE Tablets

A sustained curcumin release was measured from the PCBAE prodrug tablet formulation over approximately 16 h, while for 90:10 CMA:PEG400DA hydrogel films, curcumin release was observed over 26 h (Figure 2), which is a suitable release time for colon-specific drug delivery. Moreover, the PCBAE prodrug tablet had a continuous curcumin release with no lag phase at the start, while the as-prepared hydrogel film did not show measurable curcumin release until approximately 12 h. These differences in release characteristics are primarily a result of the differences in the characteristic size scale of the microparticles (on the order of 40 microns) as compared to the hydrogel film (approx. 350 microns). Smaller gel particle size leads to potentially faster swelling due to the higher surface to volume ratio. PBAE hydrogels typically undergo bulk erosion, which is not a size-dependent phenomenon [38,39]. However, the effect of degradation products on the release and degradation of hydrogels has been well studied, and in the case of samples with larger dimensions, the degradation products leach out much more slowly as compared to samples with smaller dimensions [38,40]. The effect of particle size on the degradation of poly(lactic-co-glycolic acid) (PLGA) microspheres has been studied, where the diffusion of degradation products from the particles affected the degradation [41,42]. In our system, it is possible that the slow leaching of curcumin-containing degradation products from the hydrogel film leads to an increase in the concentration of these products in the film, slowing the film degradation, which leads to a lag in the curcumin release during gel film degradation.

### 3.2. Superior Release Profile for PCBAE Tablets Compared to Free Curcumin Tablets

A much higher M_t_/M_∞_ curcumin release value was obtained for the PCBAE prodrug tablet as compared to the free curcumin tablet (refer: Figure 2). A sustained curcumin release is observed from the prodrug tablets, while dumping over a short period was encountered with the free curcumin tablet. For the free curcumin tablet, ~80% of the curcumin precipitates as crystalline solid, leading to its poor solubility and uptake [43]. This poor solubility along with rapid first pass metabolism has resulted in rather poor outcomes in clinical trials [44]. For instance, from studies by Sharma et al. and Garcea et al. on patients with colorectal cancer, the results showed that a very small concentration of curcumin was present in plasma and urine, which led to the lower efficiency of curcumin in the treatment of the condition under study [45,46,47,48]. Curcumin has low aqueous solubility due to its hydrophobic and crystalline nature. The incorporation of curcumin in PCBAE hinders the formation of crystals and improves solubility as curcumin dumping is avoided, due to the controlled release characteristics of the hydrogel. Furthermore, the aqueous solubility of curcumin is increased by the PCBAE degradation products.

The release of the original or native form of curcumin was confirmed by HPLC for the initial (T = 0) tablets, as well as for the tablets stored under controlled conditions (see Figure 3). Curcumin monoacrylate and curcumin diacrylate were also detected in the HPLC eluent. These species were formed from the hydrolysis of partially reacted curcumin triacrylate and curcumin diacrylate, respectively. Curcumin released in all the dissolution studies retained its antioxidant activity, as confirmed by TEAC analysis (see Figure 11).

### 3.3. Storage Stability Assessment of PCBAE Tablets

The storage stability of the tablets was studied to understand the role of excipients in the long-term storage behavior of the tablet formulations. The stability study of PCBAE prodrug tablets at standard conditions indicated that after one week, the PCBAE polymer within the tablet was partially degraded, as evidenced by the reduction in the final release time obtained; the extent of degradation during storage continued to increase with longer storage times and higher moisture absorption (see Figure 5 and Figure 6). For standard conditions, the final release time (i.e., total tablet degradation time) did not coincide with the tablet disintegration time (2 h), which shows that the degradation followed a similar hydrolytic process as with the control prodrug tablet. For the tablets stored at accelerated storage conditions, the partial PCBAE degradation observed in 1 week was comparable to the partial PCBAE degradation observed in 3 weeks at standard conditions (see Figure 6). This enhanced rate of PCBAE degradation in the tablets correlated with the higher moisture absorption at these conditions (see Figure 5), which led to the enhanced hydrolytic degradation of the gel. In addition, higher temperatures expedited the hydrolysis process [49]. For the accelerated conditions, tablet disintegration times and final release times overlapped. Since one week was enough to degrade the gel present in the tablet, it suggests that the delay in the release for longer storage times at accelerated conditions was likely to be due to a delay in the tablet disintegration. This observed delay in tablet disintegration resulted in an enhanced diffusional barrier to curcumin release, meaning that the final release time depended on the tablet disintegration time. This mechanism was confirmed by studying the dissolution of curcumin from crushed tablets, which verified that all the gel particles had degraded after 1 week of storage at the accelerated condition (see Figure 8).

### 3.4. Effect of Inherent Excipient Moisture on PCBAE Tablets

Tablet stability was explored in detail by studying the effect of storage conditions and temperature on the PCBAE prodrug tablet. The tablets underwent degradation when stored at high temperatures for 2 weeks, even in the apparent absence of environmental moisture (see Figure 9). However, the gel films (without tablet excipients) were stable at the same anhydrous conditions (see Figure 10). Since the only difference between the gel film and tablets was the MCC (binder) used for making the tablets, it is believed that the MCC is the source of water inducing hydrolysis [50,51]. MCC has an equilibrium moisture of about 3-5% under ambient conditions. It shows that the small amount of moisture present in the MCC can cause hydrogel degradation at high temperatures. Based on this evidence, using excipients with low equilibrium moisture content and packaging, such as induction sealed bottles with desiccant or blister packaging, would be recommended for the development of PCBAE-based oral formulations.

From Figure 9, it was observed that the tablets were stable for at least 2 weeks at 25 °C/low humidity conditions and that they had a release profile similar to the initial tablets. However, the tablets exhibited PCBAE degradation when incubated at 40 °C for 2 weeks at low humidity and showed complete release within 4 h. The tablets underwent degradation in 2 weeks at standard storage conditions. This shows that higher moisture and higher temperatures both cause the degradation of tablets, although high temperature (40 °C) affected the tablet stability to a greater extent than high moisture (RH = 57%).

## 4. Conclusions

Tablet formulations based on PCBAE hydrogel microparticles were developed and tested for curcumin release profiles. PCBAE provides a novel approach for potential colon-specific drug delivery, which is dependent on the pH of the GI tract. This formulation was able to achieve a sustained curcumin release of about 16 h, which was confirmed by HPLC analysis and antioxidant activity measurements. PCBAE prodrug tablets improved the solubility of curcumin by 2.5 times compared to the free curcumin tablets. The tablet stability studies exhibited a step wise change in the degradation of the prodrug tablet with an increase in storage time. The stability studies demonstrated that the small amount of moisture (~3–5 %) present in MCC can lead to PCBAE degradation in the formulation at high temperatures. While the tablets were stable at room temperature, a low moisture environment was necessary when storing these systems. The stability can be further improved by using excipients that have lower equilibrium moisture content. This approach can also be expanded to other drugs that have hydroxyl groups present in their molecular structure.

## 5. Materials and Methods

### 5.1. Materials

Curcumin was purchased from Chem-impex International, Inc. (Wood Dale, IL, USA). Acryloyl chloride, triethyl amine, 4,7,10-trioxa-1,13-tridecanediamine (TTD) and magnesium stearate were all purchased from Sigma-Aldrich (St. Louis, MO, USA). Poly(ethylene glycol) 400 diacrylate (PEG400DA) was purchased from Polysciences (Warrington, PA, USA). Avicel^®^ PH-102 (MCC) was obtained from FMC BioPolymer (Mobile, AL, USA). Sodium dodecyl sulfate, sodium bromide and sodium chloride were all obtained from Fisher Scientific (Waltham, MA, USA). All organic solvents were purchased from Pharmco-AAPER (Brookfield, MA, USA). Molecular sieves were added to all solvents to remove any trace moisture present.

### 5.2. Synthesis of Poly(Curcumin Β Amino Ester) (PCBAE) Hydrogel Films

**Figure 12:**
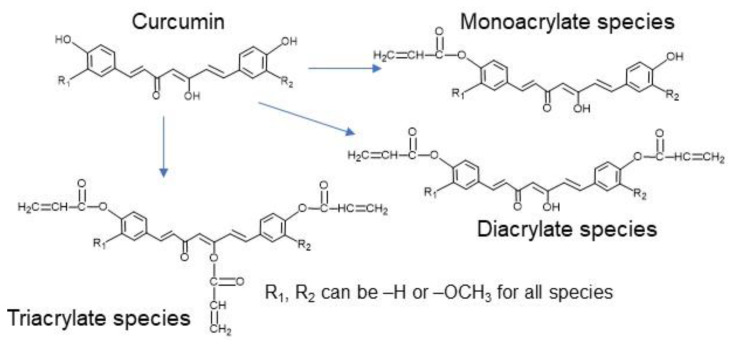
Chemical structure of curcumin and curcumin acrylates formed after the reaction of curcumin with acryloyl chloride.

Curcumin was functionalized with acrylate groups by reaction with acryloyl chloride with a 1:3 curcumin:acryloyl chloride ratio to form curcumin multiacrylate (CMA), according to an established protocol [35]. CMA had 1% curcumin monoacrylates, 42% curcumin diacrylates and 57% curcumin triacrylates, as determined previously [35]. The chemical structure of curcumin and curcumin acrylates is shown in Figure 12. PCBAE prodrug hydrogel films were synthesized using a Michael addition reaction involving reacting commercial diacrylate (PEG400DA) with a primary diamine (TTD), in combination with acrylate-functionalized curcumin [24]. The solvent used for the synthesis of the films was dichloromethane (DCM) and the amount used was 1.5 mL of solvent per gram of total monomer weight. The CMA:PEG400DA molar ratio of 90:10 was used for the synthesis of the films. PEG400DA was mixed with half of the total solvent amount and CMA was dissolved in the remaining half. TTD was added to the PEG400DA solution and allowed to pre-polymerize for 5 min at room temperature. The CMA solution was then added under continuous mixing and the entire mixture was transferred to a casting ring assembly that was kept at room temperature for 1 h and subsequently incubated in a convection oven at 50 °C for 24 h. The resulting films were washed in acetonitrile for 5 h on an orbital shaker (with solvent change every hour) to remove the unreacted monomers and oligomeric components, followed by drying overnight at 50 °C in a vacuum oven. The as-synthesized films had a thickness of approximately 350 microns.

### 5.3. Particle Size Analysis

The 90:10 CMA:PEG400DA PCBAE gel films were cryomilled using the SPEX^®^ SamplePrep 6770 Freezer/Mill^®^ with 1% magnesium stearate. This PCBAE gel powder containing magnesium stearate was used to make the tablets used in this study. Particle size of the cryomilled gel powder was analyzed using a Shimadzu SALD-7101 nanoparticle size analyzer, with particles suspended in phosphate buffered saline (PBS). PBS was used as a blank to cancel the background signal. The powder suspension was stirred continuously using a built-in plate during the analysis.

### 5.4. PCBAE Prodrug Tablet Synthesis

The blend used for tableting was prepared by mixing MCC and 90:10 CMA:PEG400DA PCBAE prodrug gel powder. The formulation details are provided in Table 1. MCC was used as a binder and diluent. This mixture was then compacted using a mortar and pestle and screened prior to use. The tablets were prepared by directly compressing the prepared blend in a tablet die (10 mm diameter) using a single station hydraulic press at 42.5 MPa. All the tablets had an approximate weight of 400 mg. All the blends were prepared as needed at a bench top scale. Since these tablets were developed for extended drug release and PCBAE hydrogel undergoes swelling in aqueous media, additional disintegrant or superdisintegrant was not added to this formulation. The primary aim of this study was to provide proof of concept for the use of PBAE hydrogels in oral solid dosage forms with good compressibility. The current formulation provided functionality and good tablet compressibility.

In addition to the prodrug (i.e., PCBAE powder containing) tablets, the tablets based on free curcumin were prepared as a control to compare drug release and storage stability. In these controls, along with curcumin, 0:100 CMA:PEG400DA PCBAE gel powder was used as an inactive component to mimic the components of the prodrug tablet. The free curcumin tablets were formulated from MCC (79% by weight), as-received curcumin powder (10.5% by weight matching the curcumin content of prodrug tablets) and cryomilled powder of 0:100 CMA:PEG400DA PCBAE (9.5% by weight) using the same method as described above. The formulation for both tablets is presented in Table 1.

### 5.5. Tablet Dissolution and Curcumin Release

The dissolution studies were completed in a USP (United States Pharmacopoeia) apparatus II in PBS at 37°C, with an impeller speed of 100 RPM. PBS of pH 7.4 with 0.1% (*w*/*w*) sodium dodecyl sulfate (SDS) was used as the dissolution media. SDS was added to PBS in alignment with the United States Food and Drug administration (USFDA) recommendation for dissolution testing of sparingly soluble drugs [52]. Then, 1 mL aliquots were taken, and the reservoir was replenished with 1 mL of fresh dissolution media for each sample time point. The sample aliquots were stored at −20 °C until further analysis. All tablet and gel degradation studies were carried out using this method. All aliquots collected during the drug release were analyzed by UV–Visible spectrophotometry (Cary^®^ 50 UV spectrophotometer). Selected aliquots from the PCBAE tablet release and aliquots from the final release data point for the stability samples were analyzed using reverse phase high-performance liquid chromatography (HPLC; Waters Phenomenex C18 Column, 5 µm, 250 mm (length) × 4.6 mm (I.D.) on a Shimadzu Prominence LC-20 AB HPLC system). The wavelength used for the detection of curcumin in both UV–Vis and HPLC was 420 nm, which is the peak absorbance wavelength for curcumin. The curcumin release data are presented as M_t_/M_∞_, where M_t_ is the absorbance at 420 nm for time t and M_∞_ is the absorbance corresponding to the theoretical curcumin loading for a given sample. In the analysis of samples using HPLC, a gradient method file from 50/50 acetonitrile/water to 100/0 acetonitrile/water over 13 min at 1 mL/min was used for all the samples with an injection volume of 50 µL. Total curcumin release quantities were determined from the HPLC chromatograms based on a corresponding calibration curve of peak area versus curcumin concentration. All the data values presented are for n = 3 with mean and standard deviation.

### 5.6. Antioxidant Activity Using TEAC Assay

The antioxidant activity of the released curcumin was evaluated using the standard Trolox equivalent antioxidant activity concentration (TEAC) assay [36]. The 2,2′-azinobis-(3-ethylbenzothiazoline-6-sulfonate) (ABTS) cation radical solution was prepared by reacting ABTS and potassium persulfate overnight. The absorbance intensity of the cation radical solution was decreased to 0.4 by dilution with PBS. Using a 96-well microplate, 10 µL of sample to be analyzed was introduced to each well, followed by 200 µL of cation radical solution. The absorbance was measured after 5 min at 734 nm and compared against the standard Trolox curve. For the TEAC assay, a decrease in absorbance was observed as the antioxidant concentration increased.

### 5.7. Standard and Accelerated Storage Stability

The stability of the tablets was studied under the standard and accelerated storage conditions as defined in USP <1150>. The recommended standard conditions are 25 °C and relative humidity (RH) of 57%, while the recommended accelerated conditions are 40 °C and 75% RH. These conditions were maintained in an air tight chamber, with beakers containing saturated salt solutions of sodium bromide and sodium chloride, respectively [53]. Different sets of tablets were stored at both conditions for 1, 2, 3, and 4 weeks. To assess the worst-case stability scenario, the tablets were stored under open conditions without any packaging, resulting in direct exposure to the moisture in the chamber. Moisture absorption was assessed, and the tablet dissolution profile was measured for each distinct exposure history.

## Figures and Tables

**Table 1:** Formulation table for poly(curcumin β-amino ester) (PCBAE) prodrug tablet and free curcumin tablet.

Ingredients	PCBAE Prodrug Tablet		Free Curcumin Tablet	
	% *w*/*w*	mg/Tablet	% *w*/*w*	mg/Tablet
90:10 CMA:PEG400DA PCBAE (prodrug microparticles) ^a^	20.0	80.00	NA	NA
Curcumin	NA	NA	10.5	42.16
0:100 CMA:PEG400DA gel powder	NA	NA	9.46	37.84
MCC	79.0	316.0	79.0	316.0
Magnesium stearate	1.0	4.000	1.0	4.000
Total	100.0	400.0	100.0	400.0

^a^–Curcumin multiacrylate (CMA); corresponding curcumin loading of 10.54% *w*/*w* and 42.16 mg/tablet.

## Data Availability

The date has been included in this manuscript.

## Supplementary Materials

The following supporting information can be downloaded at: https://www.mdpi.com/article/10.3390/gels8060337/s1, Figure S1: Curcumin release profiles for 50:50 CMA:PEG400DA poly(curcumin β-amino ester) (PCBAE) hydrogel film degradation in simulated gastric fluid (SGF) (pH 1.5) and PBS (pH 7.4). A slower release is observed at lower pH. PCBAE sample degraded completely in ~10 h at pH 7.4, while it degraded in ~21 h at pH 1.5. The degradation of PCBAE is slower at lower pH since the carboxylic acids are formed by the hydrolysis of PCBAE.
