# Supplementary material for: Poly(curcumin β-amino ester)-Based Tablet Formulation for a Sustained Release of Curcumin
Source: Gels. 2022 May 30;8(6):337. doi: 10.3390/gels8060337 (PMC9222275; doi:10.3390/gels8060337)
Supplement: Supplementary file 1 [file gels-08-00337-s001.zip › gels-1674036-supplementary.pdf]

Supplementary Material:

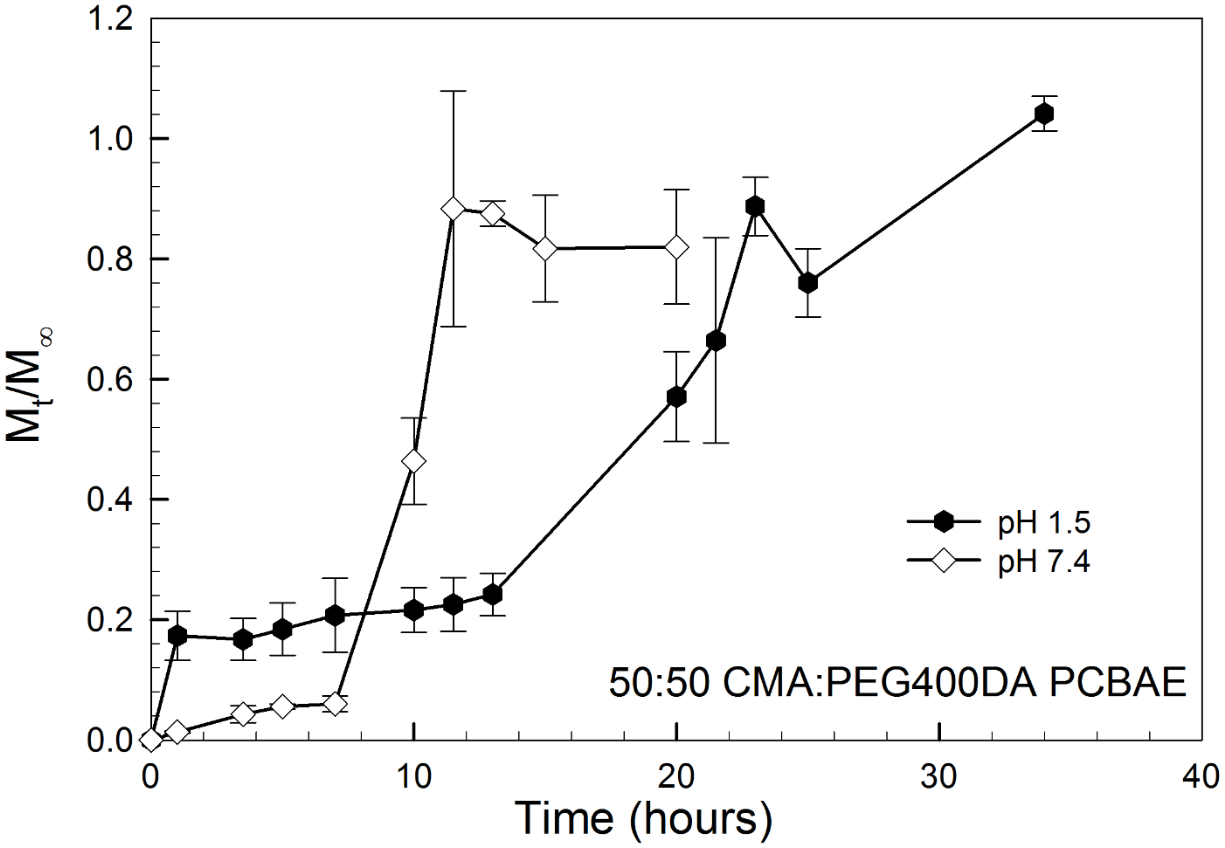

Figure S1. Curcumin release profiles for 50:50 CMA:PEG400DA Poly(curcumin β-amino ester) (PCBAE) hydrogel film degradation in simulated gastric fluid (SGF) (pH 1.5) and PBS (pH 7.4). A slower release is observed at lower pH. PCBAE sample degraded completely in ~ 10 hours at pH 7.4 while it degraded in ~21 hours at pH 1.5. The degradation of PCBAE is slower at lower pH since the carboxylic acids are formed by the hydrolysis of PCBAE. .
